# Supplementary material for: The Effects of High-Intensity Power Training versus Traditional Resistance Training on Exercise Performance
Source: Int J Environ Res Public Health. 2022 Jul 31;19(15):9400. doi: 10.3390/ijerph19159400 (PMC9367759; doi:10.3390/ijerph19159400)
Supplement: Supplementary file 1 [file ijerph-19-09400-s001.zip › ijerph-1811065-Supplementary.pdf]

International Journal of
Environmental Research and Public Health

MDPI

Supplementary Table S1. Shapiro-Wilk test of all variables.

Assessments	Group	Time	Shapiro-Wilk test		
			Statistics	df	p-value
age (year)	HIPT	-	0.798	12	**0.009** *
	TRT	-	0.942	9	0.605
weight (cm)	HIPT	-	0.882	12	0.092
	TRT	-	0.735	9	**0.004** *
weight (kg)	HIPT	-	0.868	12	0.062
	TRT	-	0.987	9	0.991
BMI (kg/m^2)	HIPT	-	0.963	12	0.822
	TRT	-	0.959	9	0.793
bench press (N)	HIPT	pre	0.963	12	0.832
		post	0.911	12	0.222
	TRT	pre	0.850	9	0.075
		post	0.802	9	**0.022** *
vertical jump (cm)	HIPT	pre	0.904	12	0.178
		post	0.894	12	0.132
	TRT	pre	0.982	9	0.974
		post	0.976	9	0.942
peak power (W)	HIPT	pre	0.960	12	0.790
		post	0.891	12	0.120
	TRT	pre	0.871	9	0.126
		post	0.867	9	0.115
mean power (W)	HIPT	pre	0.861	12	0.050
		post	0.851	12	**0.038** *
	TRT	pre	0.925	9	0.437
		post	0.980	9	0.963
peak power/kg (W/kg)	HIPT	pre	0.880	12	0.088
		post	0.909	12	0.209
	TRT	pre	0.940	9	0.582
		post	0.985	9	0.984
mean power/kg (W/kg)	HIPT	pre	0.970	12	0.910
		post	0.957	12	0.739
	TRT	pre	0.802	9	**0.021** *
		post	0.871	9	0.125

* indicated significantly non-normal according to Shapiro-Wilk tests.
